# The mechanism of histone modifications in regulating enzalutamide sensitivity in advanced prostate cancer

**DOI:** 10.7150/ijbs.109638

**Published:** 2025-04-13

**Authors:** Zhite Zhao, Yuming Jing, Zhicheng Xu, Hongfan Zhao, Xinglin He, Tong Lu, Jianhui Bai, Weijun Qin, Lijun Yang

**Affiliations:** 1 Department of Urology, Xijing Hospital, Fourth Military Medical University, Xi'an, Shaanxi 710032, China.; 2 The Second Clinical Medical College, Shaanxi University of Chinese Medicine, Xi'an, Shaanxi 710000, China.; 3 Department of Urology, Joint Logistics Support Force, Hospital 987, Baoji, China.

**Keywords:** enzalutamide, drug resistance, histone modifications, prostate cancer

## Abstract

Prostate cancer (PCa) is the second most common malignant tumor in men worldwide, particularly castration-resistant prostate cancer (CRPC), for which enzalutamide (Enz) resistance is of particular concern. Modifications to histone methylation and acetylation patterns are closely associated with resistance to Enz in these patients. As PCa progresses, cancer cells alter their histone modification patterns, leading to a reduction in Enz treatment efficacy. Signaling pathways in the tumor microenvironment regulate gene expression by affecting the activity of histone-modifying enzymes, further affecting the efficacy of Enz. This review summarizes recent research about changes in histone modification patterns that occur in drug resistance-related genes at different stages of PCa and explores the potential use of combination therapies for reversing this process, providing insights into novel treatment strategies to improve the clinical efficacy of Enz.

## Introduction

Prostate cancer (PCa) is the second leading cause of cancer-related deaths in men globally. According to a report published in *The Lancet*, global PCa cases are expected to double from 1.4 million in 2020 to 2.9 million by 2040, while Pca-related deaths are predicted to increase by 85%, from 375,000 in 2020 to nearly 700,000 by 2040 1. The androgen receptor (AR)-signaling pathway plays a crucial role in the development of PCa 2. An AR is a ligand-dependent nuclear transcription factor that can bind to testosterone or dihydrotestosterone, activating transcription of AR-responsive genes and promoting proliferation and survival of prostate cells 2. Compared to benign prostatic hyperplasia, AR expression is upregulated in primary PCa, and its expression continues to increase as the disease progresses to castration-resistant prostate cancer (CRPC) during androgen deprivation therapy (ADT) 3. AR inhibitors are important therapeutic agents for optimizing treatment plans and improving patient prognosis. In PCa, AR inhibitors mainly block the androgen signaling pathway, thereby reducing the expression of AR target genes and inhibiting the proliferation and survival of PCa cells 4. In addition, emerging proteolysis-targeting chimera (PROTAC) technology promotes degradation of the AR protein, further reducing its role in PCa and providing a new strategy for treatment of PCa 5.

Since 2012, enzalutamide (Enz) (MDV3100, Xtandi®), a nonsteroidal second-generation anti-androgen drug, has been approved by the Food and Drug Administration for the treatment of CRPC 6-7. Enz primarily exerts its effects through three AR-dependent mechanisms. First, it binds to the ligand-binding domain of AR, preventing AR activation. Second, it inhibits translocation of AR into the nucleus. Third, it inhibits the transcription of target genes by preventing AR from binding to DNA 8. Certain AR-independent signaling pathways, including the PI3K-AKT 9, NF-κB 10, Wnt 11, and glucocorticoid pathways 12, are also affected by Enz and play an equally important role in exerting anti-tumor effects and extending patient survival. Clinical studies have shown that Enz use can significantly extend the overall survival of patients with CRPC. However, approximately 30% of patients develop resistance to Enz, with an increased incidence of neuroendocrine prostate cancer (NEPC) 13-14. Unfortunately, the regulatory mechanisms underlying the development of Enz resistance remain unclear. Therefore, a deeper understanding of the mechanisms underlying this resistance is crucial for developing more effective treatment strategies.

Recent studies have indicated that histone modifications play a crucial role in the epigenetic regulation of cancer cells and potentially contribute to Enz resistance in PCa 15-16. Histone modifications, including methylation, acetylation, and phosphorylation, affect chromatin structure and regulate gene expression, impacting the proliferation, differentiation, and apoptosis of cancer cells 17-18. High-throughput analyses of Enz-resistant CRPC cells have shown that abnormalities in the activity of histone acetyltransferases and deacetylases are closely related to drug resistance 19. Furthermore, basic research and clinical trials using multilevel research techniques, combining both genomics and proteomics, have revealed changes in histone modification patterns with complex interactions within the CRPC immune microenvironment, thus providing a new perspective for understanding drug resistance in this disease.

At different stages of PCa, cancer cells may alter their regulatory patterns of histone modifications to influence differential gene expression related to drug resistance, leading to significant differences in their responses to Enz treatment. In the early stages of PCa, histone modifications promote the proliferation and survival of tumor cells by regulating the expression of key genes 20. As the tumor progresses, these modifications change, leading to decreases in Enz efficacy 20. For instance, an increase in the activity of histone deacetylases (HDACs) may make the chromatin structure more compact, suppressing the expression of certain drug-sensitive genes and thereby causing tumor cells to develop resistance to Enz 21. Changes in histone methylation may also play a significant role in Enz resistance. Variations in the activities of histone methyltransferases (HMTs) and histone demethylases (HDMs) may lead to changes in gene expression patterns, also affecting the response of tumor cells to Enz 22. An increase in H3K27me3 may lead to a silencing of certain tumor suppressor genes, making tumor cells more susceptible to developing resistance to Enz 23.

In the more advanced stages of PCa, the complexity of histone modifications grows, leading to increasingly complicated mechanisms of drug resistance. In these patients, various signaling pathways in the tumor microenvironment regulate gene expression by affecting the activity of histone-modifying enzymes, thereby influencing the efficacy of Enz 22, 24. For example, certain cytokines may activate specific signaling pathways, which, in turn, affect the expression and activity of histone-modifying enzymes, leading to the development of drug resistance 25.

Therefore, in-depth research on the specific role of histone modifications in Enz resistance at different stages of PCa may help inform the development of new treatment strategies to overcome this drug resistance and improve the clinical efficacy of Enz. By regulating histone modification patterns, more precise and effective treatment plans may be possible for patients with PCa. Thus, this review focuses on the current state of PCa research about potential drug resistance genes, histone modification patterns of these genes at various stages of PCa, and the specific molecular mechanisms of Enz resistance. This review concludes with a discussion of potential combination therapeutic strategies aimed at reversing this drug resistance.

## Histone modifications

Chromatin has a complex and dynamic molecular structure. Heterochromatin is usually highly condensed and associated with gene silencing, whereas euchromatin is more relaxed and associated with activation of gene expression 26. The basic building blocks of chromatin are nucleosomes, which consist of two copies of four histones (H2A, H2B, H3, and H4) wrapped around approximately 147 base pairs of DNA 27. Histones are the main components of chromatin, and their long tails protruding from the nucleosome allow them to undergo covalent modifications at multiple sites. These modifications include acetylation, phosphorylation, methylation, ubiquitination, and sumoylation 27. Current research focuses mainly on two types of histone modification, methylation and acetylation, which both play important roles in regulating chromatin structure and gene expression 28-29.

Histone methylation primarily occurs on the lysine (Lys, K) and arginine (Arg, R) residues of histones. This process is catalyzed by HMTs that use S-adenosylmethionine (SAM) as a methyl donor for transferring methyl groups to specific residues on histones. Lysine residues can be mono-methylated (me1), di-methylated (me2), or tri-methylated (me3) 28, and lysine residues with different methylation states have distinct functions in gene regulation. For example, H3K4me3 is typically associated with active transcriptional promoter regions, whereas H3K9me3 is associated with silent heterochromatin regions 30-31. Arginine residues can be mono- or dimethylated (symmetrically or asymmetrically), which is usually catalyzed by protein arginine methyltransferases (PRMTs) and is associated with either gene activation or repression. Symmetric dimethylation (sDMA) is generally associated with gene activation, whereas asymmetric dimethylation (aDMA) is involved in gene repression 32.

Histone acetylation is a reversible and dynamic modification process, primarily regulated by HATs and HDACs 29. The HAT family is diverse and includes P300/CBP, GNAT, MYST, P160, PCAF, and TAFII230. These enzymes transfer an acetyl group from acetyl-CoA to the ε-amino group of histone lysine residues, neutralizing the positive charge of histones and weakening their electrostatic interactions with negatively charged DNA. As a result, the chromatin structure becomes more relaxed, increasing DNA accessibility and facilitating the binding of transcription and co-transcription factors, thereby activating gene transcription 15. In contrast, HDACs remove acetyl groups from histone lysine residues, restoring the tight binding between histones and DNA and thus compacting the chromatin structure to repress gene transcription. In mammals, HDACs are classified into four categories based on their structure and function: Class I (HDAC1/2/3/8), Class II (IIa: HDAC4/5/7/9; IIb: HDAC6/10), Class III (NAD^+^-dependent deacetylases, such as sirtuins), and Class IV (HDAC11) 33. Different classes of HDACs shuttle between the nucleus, cytoplasm, and nucleoplasm, performing various biological functions. Generally, high levels of histone acetylation are associated with increased gene expression and are typically linked to the activation of gene transcription, whereas low levels of acetylation indicate repression of gene expression 33.

In summary, histone modifications play a crucial role in regulating chromatin structure and gene expression, and abnormal changes in the patterns of these modifications may be closely related to Enz resistance in PCa. This review focuses on how histone methylation and acetylation regulate sensitivity to Enz at different stages of PCa development.

## Relationship of histone modifications to enzalutamide sensitivity in castration-resistant prostate cancer

### Histone methylation in castration-resistant prostate cancer

Research has revealed that histone methylation plays a pivotal role in the progression of CRPC and the development of Enz resistance 13. Early immunohistochemical (IHC) and tissue microarray studies showed that specific patterns of histone H3 and H4 dimethylation in low-grade PCa are strong predictive indicators of tumor recurrence 34. Compared to normal tissues, PCa tissues show decreased levels of H3K9 dimethylation, H3K9 trimethylation, and H3K4 monomethylation; however, these methylation levels are increased in resistant tumors 35. These findings highlight the key role of histone markers in PCa progression and treatment response.

During the CRPC stage, studies have indicated that methylation of H3K27, H3K4, H4K12, and H3K79 primarily affects the sensitivity to Enz through AR-dependent mechanisms, with methylation of H3K4, H3K79, and H4K12 promoting gene transcription 36-38. H3K4 is typically enriched near the promoters of genes, and its level reflects the degree of transcription 36. In C42 cells, Deng *et al.* discovered that AR preferentially binds to its response element, *ARE2*, with a high enrichment of H3K4me2 observed around *ARE2*. However, this binding is significantly weakened in the presence of Enz, which suggests that Enz can alter the methylation state of H3K4, thereby regulating the transcriptional activity of AR. Furthermore, AR influences the invasiveness of PCa through the circRNA-ARC1/miR-125b-2-3p/DOT1L signaling pathway 39. Disruptor of telomeric silencing 1-like (DOT1L) is the only enzyme that can specifically catalyze methylation of H3K79 40, and it plays a key role in promoting gene expression by enhancing transcriptional elongation and influencing genomic stability 40-41. A tissue microarray study that included 80 samples of PCa and 80 benign prostate tissues utilized IHC to assess the expression levels of H3K79me2. The results showed a higher intensity of H3K79me2 staining in PCa tissues, indicating that elevated DOT1L expression correlated with an unfavorable prognosis in PCa. Subsequent studies have demonstrated that the responsiveness of AR-positive CRPC cells, including C42B cells, cells expressing the AR variant AR-V7 like 22Rv1, and Enz-resistant cells, to DOT1L-specific inhibitors is contingent on the methylation status of K79 methylation marks on the distal enhancers of the *MYC* gene bound by AR and DOT1L 42. Lysine methyltransferase 9 (KMT9) regulates the transcriptional activity of AR target genes via H4K12me1 modifications, thereby promoting the development of drug resistance 43. Additionally, KMT9 demonstrates methyltransferase activity *in vitro*, which can influence the growth of xenograft tumors. However, its role in DNA methylation remains unclear. Studies have shown that KMT9 may participate in protein methylation in the presence of Trm112 but does not directly act on DNA 40.

H3K27 is subject to mono-, di-, or trimethylation, which generally results in gene silencing 44. In a study involving patients with metastatic CRPC treated with Enz, researchers conducted next-generation sequencing of plasma DNA and analyzed the methylation patterns in mCRPC plasma. Their findings revealed that methylation changes were marked by hypermethylation at targets of polycomb repressive complex 2 (PRC2). PRC2 facilitates gene silencing through trimethylation of H3K27 via its enzymatic component, EZH2 13. Furthermore, through ChIP-seq using H3K27ac in six primary and four metastatic PCa samples, researchers identified and characterized a somatically acquired enhancer located 650 kb from the centromere of the *AR* gene. This enhancer diminished the sensitivity to Enz 45. In the Enz-resistant CRPC cell line, C4-2B, research has indicated that expression of the histone demethylase, KDM7A, is elevated. KDM7A is capable of specifically removing methyl groups from H3K27me2, thereby altering the chromatin structure and transforming it from the tightly packed heterochromatin state to an open euchromatin state. This change in chromatin structure facilitates the expression of AR and its downstream target genes, including *PSA* and *TMPRSS2*, thereby promoting the proliferation and survival of PCa cells 46.

**Figure 1:**
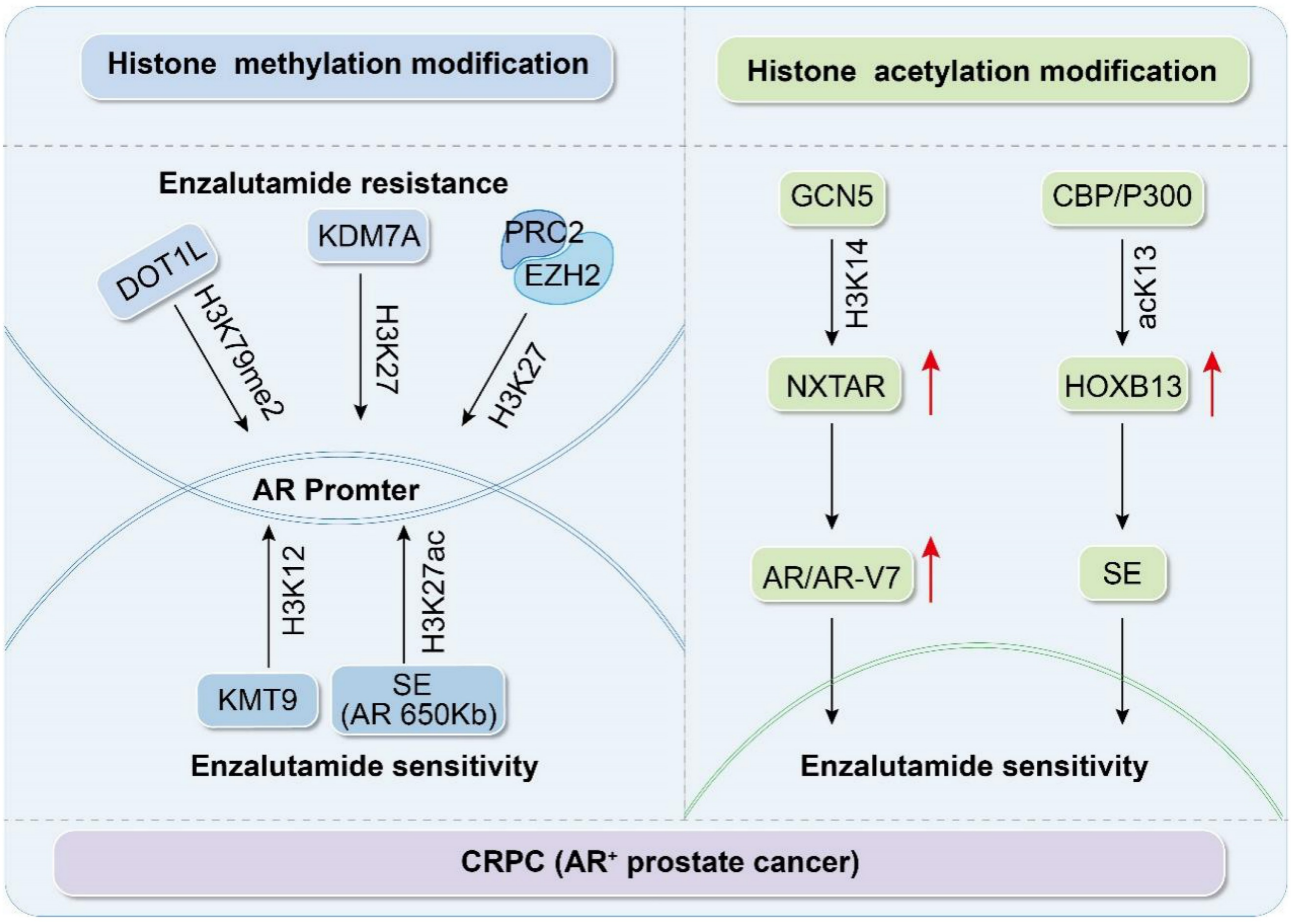
Histone methylation and acetylation participate in the AR signaling pathway to regulate enzalutamide resistance.

Comprehensive analyses have revealed that histone methylation is pivotal for mediating resistance to Enz in CRPC. Certain methyltransferases and demethylases, including DOT1L, KMT9, EZH2, and KDM7A, modulate AR activity and stability by targeting methylation at specific residues, including H3K4, H3K27, and H4K12, which, in turn, affect sensitivity to Enz (Figure 1 and Table 1). These insights not only shed light on the molecular underpinnings of PCa progression and drug resistance but also identify potential targets for innovative therapeutic approaches.

### Histone acetylation in castration-resistant prostate cancer

Histone acetylation, a key epigenetic modification, is predominantly regulated by the actions of HATs and HDACs. Typically, tumor cells display a reduced level of histone acetylation 47. Histone deacetylase inhibitors can increase histone acetylation within a cell, subsequently modifying the biological properties of tumors 48. In a study on the dominant role of AR signaling in Enz resistance, it was discovered that the novel inhibitory long noncoding RNA, *NXTAR*, is co-expressed with AR and suppressed in PCa tumors and cells. The histone acetyltransferase, GCN5, binds to and deposits the H3K14 acetylation mark, enhancing *NXTAR* expression. High *NXTAR* expression can inhibit the expression of AR and AR-V7, thereby disrupting enzyme-resistant PCa 49. Nguyen *et al.* uncovered another critical epigenetic regulatory mechanism during mass spectrometry analysis of tissue extracts from patients with CRPC. They found that acetylation of lysine 13 on HOXB13 (acK13-HOXB13), which is mediated by the histone acetyltransferase, CBP/p300, is a core regulatory factor of super-enhancers (SE) in CRPC. Notably, the binding of acK13-HOXB13 remained stable even after treatment with Enz. Furthermore, three-dimensional spheroid formation experiments showed that cells expressing wild-type HOXB13 formed significantly larger spheroids and exhibited Enz resistance, whereas cells expressing the HOXB13K13A mutant did not possess these characteristics. These findings suggest that the absence of HOXB13 acetylation affects the proliferation and sensitivity of CRPC cells to Enz. At a mechanistic level, this study also revealed that p300 protects JMJD1A from ubiquitin-mediated degradation by STUB1 via acetylation, thereby enhancing the stability and activity of JMJD1A. JMJD1A promotes transcriptional activation of tumor growth-related genes by removing the repressive histone mark, H3K9me2, thus driving PCa progression 50. This discovery not only highlights the important role of the p300-JMJD1A axis in PCa but also provides a new potential therapeutic target for CRPC, further emphasizing the key role of epigenetic regulation in tumor development.

## Relationship of histone modifications to enzalutamide sensitivity in neuroendocrine prostate cancer

**Figure 2:**
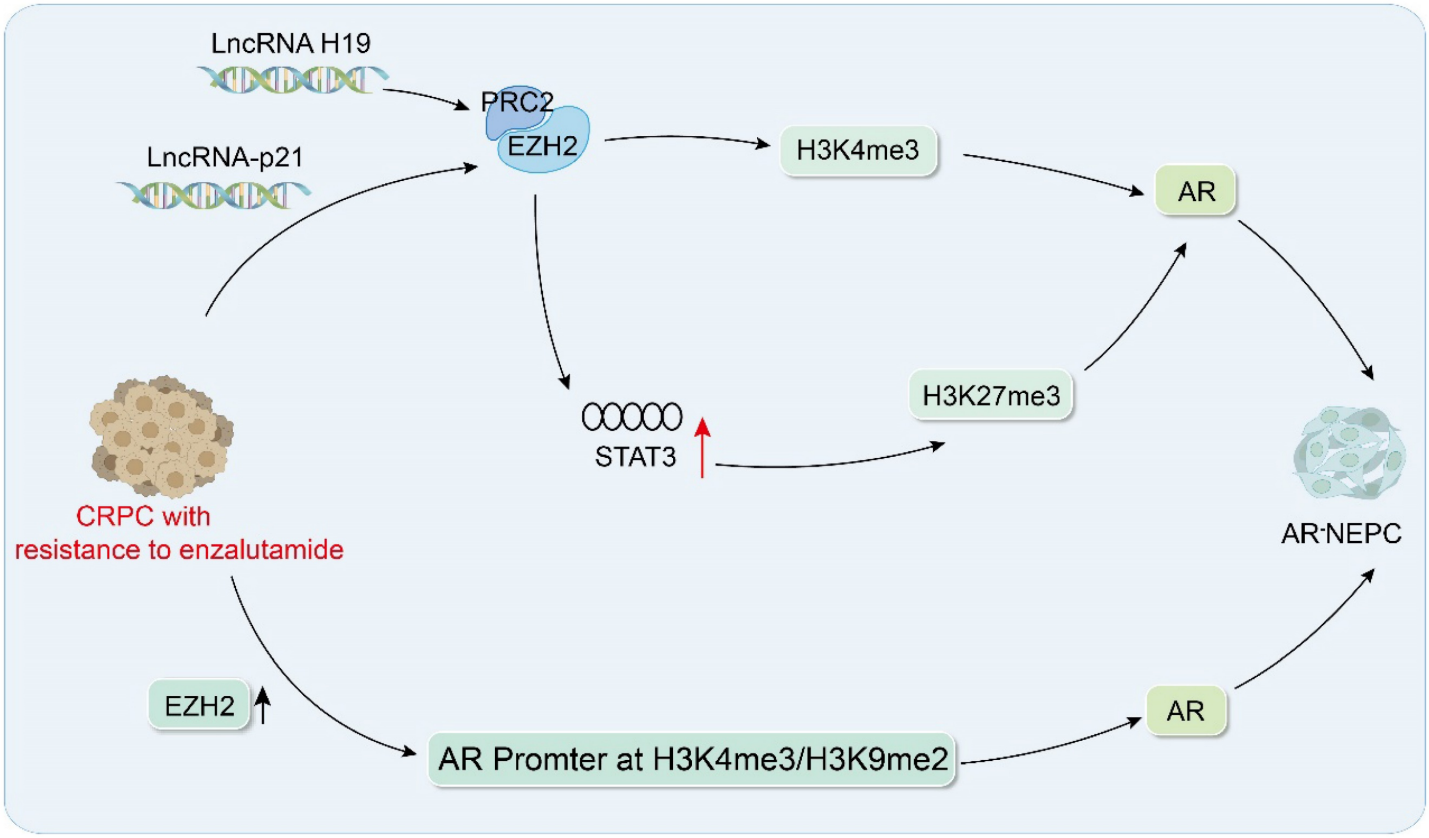
Histone modifications drive neuroendocrine differentiation in prostate cancer. AR pathway: lncRNA H19 activates AR signaling by binding to the PRC2 complex and regulating H3K27me3 and H3K4me3; lncRNA-p21 binds to EZH2 while interfering with PRC2 formation, and enhances methylation of STAT3; ENZ enhances the H3K4me3 and H3K9me2 in the promoter region of AR.

In patients with CRPC who exhibit resistance to Enz, tumors may undergo transformation to NEPC due to post-transcriptional modifications, including *AR* gene mutations 51-53. NEPC is a highly aggressive subtype of PCa characterized by resistance to hormonal therapy, poor prognosis, and limited treatment options. This type of cancer typically exhibits rapid progression and a high degree of metastasis, and patients are often diagnosed at an advanced stage. Certain histone modification pathways may contribute to NEPC progression 54. Consequently, targeted therapeutic interventions focusing on these specific epigenetic modifications may slow advancement of this disease. The long noncoding RNA, *H19*, has been shown to be markedly overexpressed in individuals with NEPC, and studies have confirmed that *H19* not only triggers differentiation of PCa into NEPC but also bolsters resistance of tumor cells to ADT. Mechanistically, *H19* binds to miR-675 to regulate expression of its downstream target genes, *RB1* and *EZH2*. In addition, *H19* interacts with PRC2 by modulating the activity of DNA methyltransferase 1 (DNMT1), thereby regulating H3K27me3 and H3K4me3 modifications, which ultimately activates the AR signaling cascade. These multi-layered regulatory mechanisms collectively drive the transition from PCa to the more aggressive NEPC phenotype 55. Luo et al. further elucidated the critical role of lncRNA-p21 in promoting NED of PCa cells under Enz treatment. Mechanistically, lncRNA-p21 directly interacts with the enhancer region of EZH2 (enhancer of zeste homolog 2), a core component of the PRC2. This interaction disrupts the binding of EZH2 to other PRC2 core subunits, such as SUZ12 and EED, thereby impairing assembly of the PRC2 complex. Consequently, the loss of PRC2 integrity leads to a significant enhancement of EZH2's methyltransferase activity, independent of its canonical PRC2-dependent functions. Moreover, lncRNA-p21 facilitates the formation of a protein complex involving EZH2, AKT, and STAT3. Specifically, lncRNA-p21 acts as a molecular scaffold, promoting the physical interaction between EZH2 and AKT, which in turn phosphorylates and activates STAT3. Through its methyltransferase activity, EZH2 catalyzes the trimethylation of STAT3 at lysine 180 (K180), a post-translational modification that stabilizes STAT3 and enhances its transcriptional activity. This hyperactivation of STAT3 signaling drives the expression of neuroendocrine markers, such as synaptophysin SYP and CHGA, thereby inducing neuroendocrine differentiation of PCa cells. Additionally, the lncRNA-p21-mediated activation of the EZH2/STAT3 axis contributes to the development of Enz resistance. The sustained STAT3 activity not only promotes NED but also upregulates anti-apoptotic and pro-survival genes, enabling tumor cells to evade AR-targeted therapies. Collectively, these findings highlight lncRNA-p21 as a pivotal regulator of NED and drug resistance in PCa, acting through the modulation of the EZH2/STAT3 signaling pathway 56. Kleb et al. conducted research using AR and AR^+^ NEPC patient-derived xenograft (PDX) models, confirming that in tumors lacking AR, the *AR* promoter is enriched with inhibitory histone modifications of H3K27me3 and H3K9me2. The study also highlighted the interplay between DNA methylation and histone modifications in AR silencing. In AR- tumors, the AR promoter is often hypermethylated at CpG islands, which synergizes with H3K27me3 and H3K9me2 to maintain a stable and heritable repressive state. This epigenetic silencing mechanism effectively abolishes AR expression, contributing to the androgen-independent phenotype observed in NEPC 57. Ku *et al.* reported that in prostate cancer with *PTEN* and *RB1* deletions and *PTEN*, *RB1*, and *TP53* deletions, catalytic inhibition of EZH2 can reactivate AR signaling, causing tumors to resensitize to Enz, while also reducing the expression of NEPC target genes. These data strongly support the importance of EZH2-mediated histone methylation in AR inhibition in NEPC 58. These findings also highlight the capacity of histone-modifying enzymes to affect post-translational modifications of AR, which are pivotal in the advancement of NEPC (Figure 2). Consequently, these enzymes are promising targets for innovative therapeutic approaches.

## Relationship of non-histone modifications to enzalutamide resistance

Although research into the relationship of non-histone modifications to Enz sensitivity is limited, existing studies suggest that a variety of mechanisms, including RNA modifications and chromatin remodeling, may play important roles. For example, it has been found that the m6A “reader” protein, YTHDF3, and the RNA-binding protein, G3BP1, can jointly regulate translation of AR mRNA. Under the acute stress of Enz-induced inhibition of the AR pathway, m6A-modified AR mRNA is transferred from actively translating polysomes to RNA-protein stress granules, thereby significantly reducing AR translation 59. Chromatin-remodeling complexes also play a key role in Enz resistance. It has been found that SMARCC2, a core component of the SWI/SNF complex, is selectively essential in Enz-resistant PCa cells. In these resistant cells, chromatin occupancy of SMARCC2 and BRG1 is significantly increased, and these regions overlap with the binding sites of transcription factors related to CRPC, indicating that they may regulate gene expression by altering the chromatin structure, thereby participating in the resistance process 60.

## Histone modification-targeted therapies

### Histone methylation-targeted therapies

EZH2 is instrumental in facilitating lineage plasticity, differentiation shifts, and in processes closely associated with NEPC development 61. The EZH2 inhibitor, tazemetostat, has been employed therapeutically during investigations of the impact of EZH2 catalysis inhibition on different PCa subtypes 62. This research has revealed that AR-positive PCa cell lines exhibit a response to treatment with 5 μm of tazemetostat over a period of 6 days. In contrast, AR-negative, NEPC patient-derived organoid models demonstrate minimal to no response when administered the same dose of tazemetostat 63. Furthermore, in NEPC PDX models, tazemetostat treatment failed to markedly influence tumor growth rates. Notably, NEPC models that are unresponsive to EZH2 inhibition have been shown to exhibit a pronounced response to the cyclin-CDK2 inhibitor, CIR7-2512 63. Moreover, research has led to the development of a therapeutic approach involving a novel, dual-action peptide, FR13. FR13 not only inhibits the methyltransferase activity of EZH2 but also blocks its positive regulatory effect on AR gene expression in PCa cells. Experimental results demonstrate that the combination therapy of FR13 and Enz significantly eradicates Enz-resistant PCa cells 64. These findings suggest that a targeted or combined therapeutic approach is necessary to address the atypical coactivator functions of EZH2. These findings also highlight the potential efficacy of EZH2 inhibitors for treatment of certain PCa subtypes but their limited efficacy for others, thus reinforcing the significance of tailoring therapeutic approaches. Several EZH2 inhibitors, including lirametostat, tazemetostat, valemetostat, PF-06821497, and SHR2554, are currently being investigated in early-phase clinical research. In Phase 1 clinical trials, PF-06821497 combined with Enz for the treatment of patients with mCRPC who had been treated with abiraterone achieved a median radiographic progression-free survival (rPFS) of 17.1 months, which was of significant benefit compared to historical data (rPFS of 4.8 months) 65.

LSD1 is a histone demethylase that is frequently overexpressed in patients with advanced PCa 66. Unlike EZH2, LSD1 has been shown to have efficacy for the treatment of both CRPC and NEPC. Research has shown that the LSD1 inhibitor, bomedemstat, exhibits significant antitumor effects in CRPC models with evidence of tumor regression observed in xenografts from patients 67-68. Bomedemstat is characterized by favorable pharmacokinetic and pharmacodynamic profiles, showing activity in a range of advanced CRPC models, including in patients with NEPC 67. Importantly, bomedemstat has also been shown to achieve substantial tumor accumulation at micromolar concentrations *in vivo* 68.

A recent clinical trial was conducted to investigate the novel LSD1 inhibitor, CC-90011. Early findings in patients with small cell lung cancer and squamous non-small cell lung cancer indicate that CC-90011 has a favorable safety profile and superior pharmacokinetic properties, suggesting it may be a promising candidate for both monotherapy and combination therapy in oncology 69.

### Histone acetylation-targeted therapies

Therapeutic interventions with HDAC inhibitors (HDACi) have been shown to restore responsiveness to taxanes, anti-androgens, and a range of chemotherapeutic agents 70-71. Furthermore, synergistic use of HDACi and taxanes has been demonstrated to halt tumor progression and enhance cell mortality 71. Research by Liu revealed that minimal doses of the HDACi, panobinostat, could resensitize PCa cells to the nonsteroidal anti-androgen, bicalutamide 72. Furthermore, belinostat (PXD101), another HDACi, downregulates AR expression, thereby preventing the emergence of CRPC *in vivo* in the context of hormonal therapy 73. HDACi have demonstrated significant antitumor efficacy when used alone or in combination. In patients with CRPC, clinical studies employing panobinostat and SB939 have indicated a decrease in PSA levels with disease stabilization, and phase II trials have already been conducted. Unfortunately, these treatments also have considerable side effects, with nausea and fatigue being the most frequently reported 74-75. Vorinostat, a standard treatment for metastatic PCa and advanced solid tumors, is often administered alongside chemotherapeutic agents like docetaxel as part of ADT. However, some patients experience severe toxic reactions, including febrile neutropenia and sepsis, particularly when vorinostat is used in conjunction with docetaxel 76-77. Curcumin, a component derived from the turmeric spice, is an alternative HDACi. It has been shown to reduce urinary tract infections in patients with PCa when co-administered with chemotherapy 78. Despite substantial advancements in epigenetic therapies involving HDACi, managing adverse reactions remains challenging, possibly due to a lack of complete specificity for HDACi. Furthermore, HDACi typically use Zn^2+^-binding groups, including hydroxamic acids, thiols, carboxylic acids, ketones, and 2-aminobenzenes, which strongly bind to other important metalloenzymes, leading to cytotoxicity and thus limiting the clinical application of these agents 79. For example, studies have shown that an anti-androgen/HDACi hybrid can be developed by combining the structural features of Enz with those of hydroxamic acid-based HDACi such as SAHA. Research has found that this hybrid is more effective in androgen-sensitive LNCaP cells and androgen-insensitive PC-3 cells than Enz alone 80.

## Summary and Outlook

Histone modifications are crucial to the progression of CRPC and the development of Enz resistance 16. In fact, certain acetylation and dimethylation patterns of the histones, H3 and H4, serve as robust predictors of low-grade PCa recurrence 13. During the CRPC phase, the methylation statuses of H3K27, H3K4, H4K12, and H3K79 affect Enz sensitivity via AR-dependent pathways. Methylation at H3K4 enhances gene transcription, whereas methylation at H3K27 results in gene silencing 36-38. Histone acetylation, governed by HATs and HDACs, is typically reduced in tumor cells but can be elevated by HDACi, thus modifying tumor biology 76-78. In patients with CRPC who become resistant to Enz, there is a potential for tumors to evolve into NEPC because of post-transcriptional changes, including *AR* gene mutations 54.

In addition, a limited number of studies have revealed the roles of histone phosphorylation and ubiquitination in Enz resistance. Histone phosphorylation, which typically occurs at sites like H3 (e.g., H3S10, H3T11) and H2AX (e.g., H2AXS139, known as γH2AX), is involved in chromatin remodeling, DNA damage repair, and cell cycle regulation 81. Research has suggested that Enz resistance may be associated with enhanced DNA damage repair capabilities in tumor cells. Histone phosphorylation may indirectly affect the AR signaling pathway by regulating AR transcriptional activity or by interacting with other transcription factors, thereby reducing the efficacy of Enz. For example, phosphorylation of histone H2AX (γH2AX) is a marker of DNA double-strand breaks, and elevated levels may promote tumor cell survival under drug pressure 82. In contrast, histone ubiquitination, which primarily occurs on histones H2A (e.g., H2AK119ub) and H2B (e.g., H2BK120ub), is involved in the regulation of chromatin structure and gene expression 83. Histone ubiquitination may influence Enz resistance by regulating the expression of AR target genes. For instance, H2AK119ub is generally associated with gene silencing and may suppress key genes in the AR-signaling pathway. Targeting these mechanisms by inhibiting histone-modifying enzymes (e.g., kinases, ubiquitin ligases, and deubiquitinases) may be a potential strategy for reversing Enz resistance. Drugs targeting E3 ubiquitin ligases or HDACs are currently being investigated 84. Epigenetic therapies targeting CRPC are predominantly aimed at more advanced disease stages, with approved agents including sipuleucel-T, docetaxel, cabazitaxel, abiraterone, alpharadin, and Enz 72. However, the most effective sequencing and combination strategies for these treatments are unknown and continue to be the subject of research.

In the future, advanced multiomic sequencing techniques to identify predictive biomarkers will be essential for identifying patients with PCa at various stages who are most likely to benefit from histone methylation-targeted therapies, as well as for tracking treatment efficacy. This approach will be vital for improving patient selection and enhancing therapeutic outcomes 85. By dynamically evaluating histone modification levels, biologically relevant gene mutations, gene expression profiles, and chromatin accessibility patterns, we may be able to more accurately stratify patients according to their likely treatment responses and to develop more tailored treatment strategies 86.

Furthermore, the synergistic effects of DNA methylation and histone modifications, which jointly govern gene expression, may play a significant role in determining sensitivity to Enz. The integration of histone modification regulation with immunotherapy represents an emerging frontier of research. Histone modifications influence the immunogenicity of tumor cells and the functionality of immune cells within the tumor microenvironment. For example, they can modulate the expression of immune checkpoint molecules on tumor cell surfaces. Adjusting the activity of histone-modifying enzymes can thus alter the expression of immune checkpoint molecules like programmed death ligand 1 (PD-L1) 87. In Enz resistance, the concurrent application of histone modification modulators and immune checkpoint inhibitors (such as anti-PD-1/PD-L1 antibodies) may bolster the immune system's surveillance and cytotoxic capabilities against tumor cells 86.

Although epigenetic therapies, whether used alone or in combination with other treatments, have demonstrated benefits, they also have adverse effects, highlighting the need to enhance the specificity of these drugs and minimize their side effects 25. Consequently, the development of natural compounds, optimization of dosing for histone-modifying drugs, and establishment of personalized treatment plans are increasingly critical for patient management**.**

## Figures and Tables

**Table 1:** Histone modifications in CRPC and their role in Enz sensitivity

Modification Type	Substrate	Site	Function	Regulation	Impact on Enz Sensitivity
Methylation	H3	H3K4 me3	Enriched near gene promoters	SMYD2↑ AR stability↑	Increased AR activity and resistance to Enz; SMYD2 inhibition sensitizes CRPC cells to Enz
Methylation	H3	H3K79 me2	Enhances transcriptional elongation	DOT1L↑	DOT1L inhibition reduces MYC gene expression and sensitizes AR-positive CRPC cells to Enz
Methylation	H4	H4K12 me1	Regulates AR-target gene transcription	KMT9	KMT9 activity promotes CRPC progression and resistance to Enz
Methylation	H3	H3K27 me3	Gene silencing; mediated by PRC2/EZH2	EZH2	Hypermethylation at PRC2 targets correlates with Enz resistance in mCRPC
Demethylation	H3	H3K27 me2	Converts heterochromatin to euchromatin	KDM7A	KDM7A upregulation in Enz-resistant CRPC cells promotes AR activity and resistance to Enz
Acetylation	H3	H3K14ac	NXTAR↑ AR and AR-V7↓	GCN5	High NXTAR expression disrupts Enz-resistant PCa
Acetylation	H3	H3K9 me2	Protects JMJD1A from degradation	p300	p300-JMJD1A axis drives PCa progression and Enz resistance
